# State of the art: radiomics and radiomics-related artificial intelligence on the road to clinical translation

**DOI:** 10.1093/bjro/tzad004

**Published:** 2023-12-12

**Authors:** Shweta Majumder, Sharyn Katz, Despina Kontos, Leonid Roshkovan

**Affiliations:** Department of Radiology, University of Pennsylvania Perelman School of Medicine, Philadelphia, PA 19104, United States; Department of Radiology, University of Pennsylvania Perelman School of Medicine, Philadelphia, PA 19104, United States; Department of Radiology, University of Pennsylvania Perelman School of Medicine, Philadelphia, PA 19104, United States; Department of Radiology, University of Pennsylvania Perelman School of Medicine, Philadelphia, PA 19104, United States

**Keywords:** artificial intelligence, deep learning, machine learning, oncologic imaging, radiomics

## Abstract

Radiomics and artificial intelligence carry the promise of increased precision in oncologic imaging assessments due to the ability of harnessing thousands of occult digital imaging features embedded in conventional medical imaging data. While powerful, these technologies suffer from a number of sources of variability that currently impede clinical translation. In order to overcome this impediment, there is a need to control for these sources of variability through harmonization of imaging data acquisition across institutions, construction of standardized imaging protocols that maximize the acquisition of these features, harmonization of post-processing techniques, and big data resources to properly power studies for hypothesis testing. For this to be accomplished, it will be critical to have multidisciplinary and multi-institutional collaboration.

## Introduction to radiomics and artificial intelligence in radiology

Radiomics leverages conventional medical imaging to extract occult digital features embedded in the image that are reflective of tissue histology, biological activity, functional properties, and more. These features can be quantitatively extracted from image regions of interest (ROI) to yield radiomic feature patterns that reflect tissue architecture and are influenced by genetic expression, tissue microenvironment, and effects of therapeutic intervention. As a result, these radiomic patterns can serve as an “imaging phenotype” of malignancy that can be used as a biomarker providing clinically significant information in a noninvasive manner. The imaging phenotype can be integrated with conventional radiologic parameters such as lesion size, presence of contrast enhancement, Fluorodeoxyglucose (FDG) avidity on PET-CT,^1^ and the presence of restricted diffusion on MRI^2^ to generate a more complete imaging description of disease.

Artificial intelligence (AI) methods are used at various stages of a radiomics pipeline. AI is an umbrella term that refers to the use of computer algorithms and machines to automatically perform intelligent tasks.^3^ Machine learning (ML) employs statistical methods to “learn” and improve from “experience” (ie, data)^4^ and can be applied to the classification and prediction of the imaging phenotype.^5^ This can be achieved through human hand-crafted imaging features or autonomously through deep learning (DL)—an automated computer architecture that uses multi-layered neural networks to map input images into desirable outputs (such as tumour segmentation, prognostic predictions, etc.).^5^ Machine learning/DL methods are of paramount importance in radiomics since they can learn from large, multi-dimensional imaging datasets to build classification models and classify new data.

**Figure 1.**
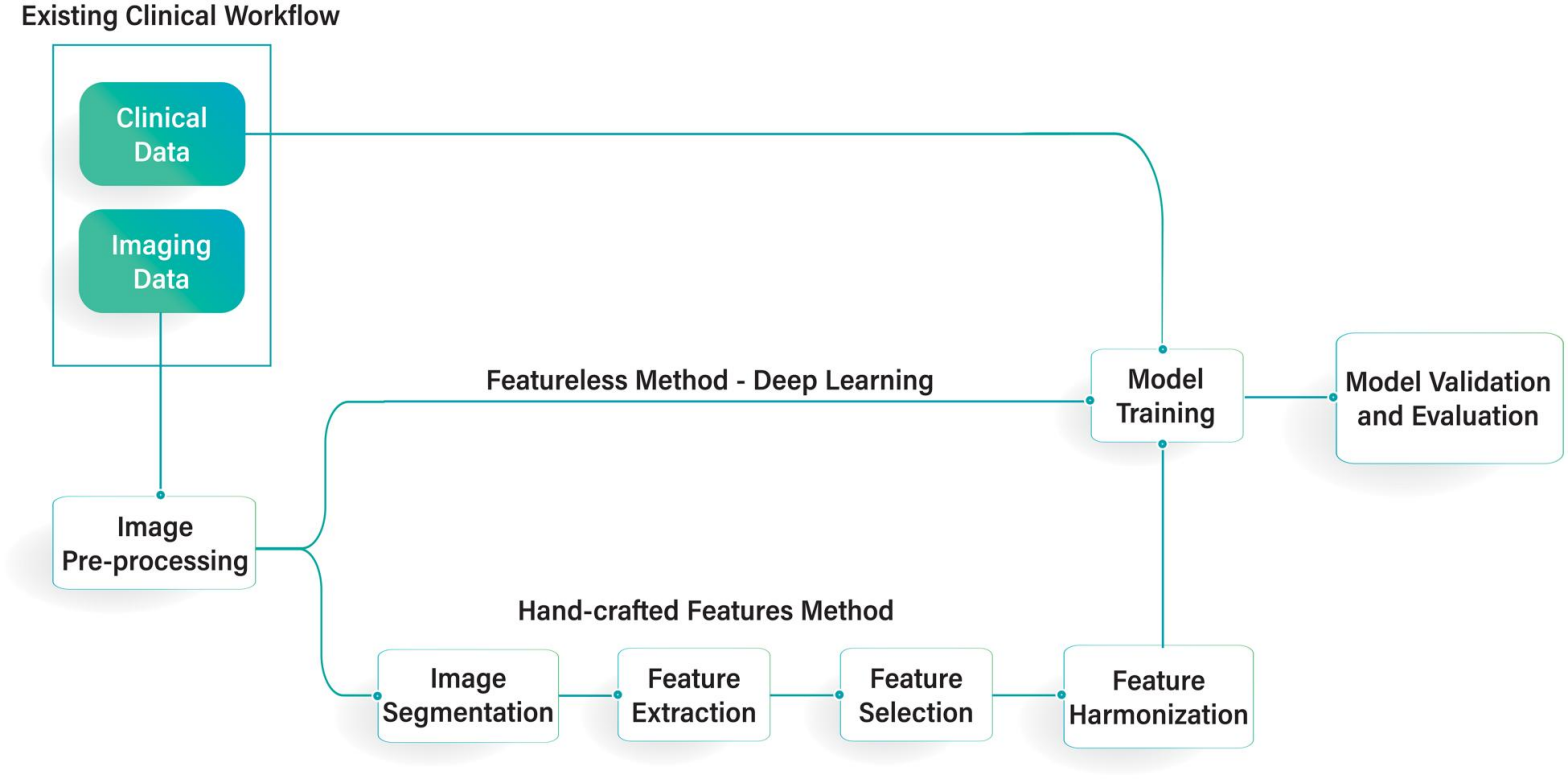
Typical radiomics workflow. The workflow starts in the clinic by collecting clinical and imaging data. The imaging data are converted to files amenable to radiomics analysis through several steps described in “Image Acquisition and Conversion to Segmentable Data, Image Pre-Processing, and “Image Segmentation.” Then the imaging data along with the clinical data go through a machine learning and/or deep learning pipeline to model specific questions about the disease such as recurrence risk.

The pipeline for radiomics, like any other quantitative imaging analysis workflow, starts with the acquisition, selection, and curation of the medical images, including tissue segmentation and quality control. Then features are either manually extracted from the segmented volume of interest (VOI) for ML methods or the VOI is directly passed through a DL network which then models features into the different target variables of clinical importance. If manual extraction is performed, then the extracted features undergo further feature-engineering methods, such as feature selection, to avoid overfitting data by the modelling algorithm. Figure 1 delineates the workflow process of a typical radiomics analysis.

### Image acquisition and conversion to segmentable data 

Radiomic analysis can be performed on any medical imaging data including CT, MRI, ultrasound, and PET. In order to ensure a high-quality analysis, standardized imaging acquisition protocols can reduce unwanted technical sources of variability including kernel reconstruction, timing and dose of intravenous contrast, time to repetition and time to echo on MRI, and radiotracer uptake time and dose on PET.^6^ The downloaded image data are taken from the Picture Archiving and Communication System and converted to a segmentable file format to be used with segmentation software such as ITK-SNAP.^7^

### Image pre-processing

There is non-biological variability, termed “batch effects,”^8^^,^^9^ present in medical imaging. These can be due to differences in scanner hardware and software, heterogeneity in imaging acquisition protocols, and technical image artefacts such as motion and can affect radiomics evaluation of the disease. While it is currently not possible to eliminate all technical variabilities, those introduced by a specific scanner at a specific site can be limited by comparing image variability on phantom studies.^10^ In contrast, variabilities introduced by differences in acquisition sites, scanners, and parameter studies can be addressed through harmonization on the image domain (image pre-processing) and the feature domain (“Feature Harmonization”). Computational methods of image pre-processing include:

1. Image resampling: Resampling to homogenize the image resolution^11^ across multiple scans.
2. Normalization: Homogenize signal intensities which are arbitrary and filter outliers^12^ across multiple scans.
3. Discretization: Group pixels into bins of similar intensity ranges.^11^
4. Bias field correction: Homogenize spatial signal variation (for MRIs).^11^^,^^13^

### Image segmentation

Medical images are segmented via manual, automatic, or semi-automatic segmentation methods to determine the 2D ROI or 3D VOI.^14^ Manual segmentation refers to human expert delineation of the tumour volume while automatic segmentation is performed by algorithms. In semi-automatic segmentation, users aid the software in determining segmentation parameters such as tissue margins and image window selection.^15^ Examples of semi-automatic segmentation methods include region-growing methods (ie, GrowCut on 3D Slicer [www.slicer.org]), feature space methods (ie, Multichannel Markov Random Field Framework)^16^ and annotation tools such as graph cut, level-set, active contours.^17^ Along with requiring user input, these methods can be limited by the nature of the imaging data. For example, GrowCut works best with homogenous and bright CT scans when segmenting lung tumour.^18^

In contrast, fully automated methods do not require user interactions and typically involve DL-based methods to train models on labelled medical images or “training dataset” and subsequently apply those models on an experimental dataset.^19^ Since, DL-based methods use convolutional neural networks (CNNs), the deterministic nature of their output can avoid intra- and inter-observer variabilities.^20^

### Hand-crafted feature curation

#### Feature extraction

**Figure 2.**
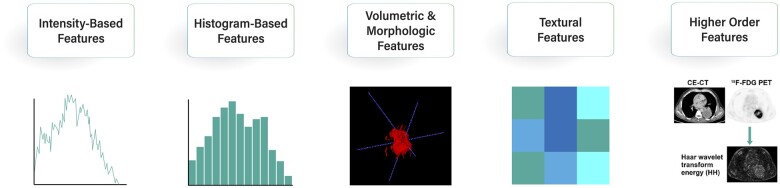
Different types of hand-crafted features. (From left to right) (i) intensity-based features are related to statistics of the grey-level intensity of each voxel; (ii) histogram-based features are related to the statistics of intensities after grouping them into bins; (iii) volumetric and morphologic features describe the volume and shape of the VOI (image generated using ITK-SNAP^7^); (iv) textural features relate the spatial relationship of the grey-level intensities; and (v) higher-order features are extracted after transforming the image through filters (image from Ref. 21).

The image ROIs/VOIs contain numerous features which can be extracted to construct an imaging phenotype. Currently, radiologists provide qualitative assessments and simple quantitative lesion measurements such as lesion size, attenuation (CT), or signal intensity (MRI). Through radiomic analysis these features can be quantitated and classified.^17^ The commonly used radiomic features include (see Figure 2):

1. Intensity- and histogram-based features: first-order statistical features that model the voxel intensities. Intensity-based features describe the distribution of the grey levels in the ROI and histogram-based features are extracted after grouping intensities into bins (discretization). These features are not concerned with the spatial relationship of intensities.^22^^,^^23^
2. Volumetric and morphologic features: associated with the VOI and the shape and geometry of the ROI.^22^
3. Texture-based features: second-order statistics which quantify the spatial arrangement of the voxel intensities.^24^ These include grey-level run length matrix (GLRLM), grey-level co-occurrence matrix (GLCM), grey-level size zone matrix, neighbouring grey tone difference matrix, local binary pattern, and use matrices which quantify the textural variations based on the spatial arrangements of the grey-level intensity.^25^
4. Higher-order features: extracted after using filters such as Gaussian filter and Gabor filter and are used to capture complex patterns in the data^25^ or extracted using artificial neural networks (“Deep” features).^6^

During feature extraction, factors other than image-processing parameters that influence the feature values for different feature families should be accounted for including grid distance (used in textural features), feature aggregation (aggregation of different values for one feature into a single value), and distance weighting (emphasizes local intensities; used in PyRadiomics).^23^^,^^26^

#### Feature harmonization

Feature harmonization is often used to create a homogenous set of features when using images acquired from different scanners.^8^^,^^9^ Harmonization can adjust for undesirable variations within an imaging dataset or “batch” generated during image acquisition.^27^ The concept of batch-effect removal comes from genomics, where adjusting batch effects across multiple datasets for microarray gene expression is necessary.^9^^,^^27^ Some commonly used methods employed for radiomic analysis include^27^ location-scale, matrix factorization, and discretization methods. For small datasets, ComBat, an empirical Bayes method,^9^ is particularly valuable for feature-level harmonization.^21^

ComBat is a location-scale method using Bayes estimations for mean and variance of features in each batch to transform them into a unified mean and variance.^27^ This technique has been implemented in multiple languages (ie, R, Python, and MATLAB)^28^ and has shown promising results in mitigating batch-effect-induced differences in radiomic images in lung cancer.^22^ Limitations of ComBat include an assumption that technical errors are normally distributed and the fact that only a single batch-effect can be corrected at a time.^29^ Several methods are being tested to address these limitations including a Gaussian Mixture Model ComBat for improved batch-effect correction and a Nested ComBat method for multiple batch-effect correction.^29^

#### Feature selection

Since not all of the numerous features extracted are useful, feature selection is the next important step for building a robust and generalizable radiomics model.^11^ Having too many features, called “high dimensionality,” can lead to model overfitting which might not work for new data not previously evaluated by the model.^30^ Through feature selection, the number of features is reduced to create a more robust and reproducible signature by considering two factors—feature stability and feature redundancy. Feature stability refers to the robustness of the imaging feature to training sample set variability. Feature redundancy refers to reducing the redundant features identified by being highly correlated to or similar in what they characterize^17^ since these are of little value.

There are multiple approaches to assess feature stability based on available imaging data for comparison. If test-retest dataset (same image from the same patient and scanner obtained a few minutes apart) is available, then the intra-class correlation coefficient (ICC)—an index from 0 to 1 that reflects test-retest agreement—can be calculated for each feature.^31–33^ If multiple phantom images for the same and different scanners can be acquired,^20^ then the concordance correlation coefficient (CCC) and dynamic range (DR) for each feature^34^^,^^35^ can be calculated. Features with higher CCC, DR, and ICC (ie, CCC and DR > 0.9, ICC > 0.75) are considered to have higher test-retest/inter-observer agreement, biological range, and good reproducibility^35^^,^^36^ and are considered stable with unstable features removed using the cutoff. Furthermore, if multiple image segmentations are available (same image, different radiologist or algorithm), the ICC for the features across the different annotations can be evaluated.^11^^,^^20^

Once feature stability has been assessed, the next step is to eliminate redundancy in feature sets. This can be done through supervised (requires labels) or unsupervised (does not require labels)^5^ methods. As a first step in a supervised approach, a pairwise correlation test can be performed to remove the features with high correlation^37^ and be followed by further supervised feature selection using filter, wrapper or ML-based methods. Correlation clusters and heatmaps are helpful for visualizing the feature set performance derived from different selection methods when training radiomic models.^11^^,^^20^^,^^37^ Feature selection methods can also be incorporated during model building including embedded methods such as least absolute shrinkage and selection operator (LASSO). With LASSO, the model is regularized by “shrinking” the feature weights and setting weights of the non-contributing features to zero.^38^ Finally, unsupervised feature selection methods can be used to reduce feature dimensionality including clustering, t-distributed stochastic neighbour embedding, and principal component analysis.^20^

This summary of the process of feature selection highlights the variability that can be introduced by choice of the method in feature selection which has been reported in the literature^38^ including when evaluating CT images of lung cancer patients^39^ and when devising radiomic predictors of tissue histology.^37^ It is challenging to standardize feature selection methods given its dependency on the available data, and the choice of method should be made based on the imaging data characteristics of a given dataset. An understanding of the choice of feature selection method as a source of variability is important and methods should be reported in detail in radiomic studies to ensure reproducibility.^20^

### Featureless DL methods

Deep learning methods are becoming increasingly popular in different parts of the radiomics pipeline (see “Image Segmentation” for DL-based image segmentation). Featureless DL methods, which do not require hand-crafted radiomic features, are particularly interesting since they avoid additional steps of image pre-processing and feature engineering, and the resulting sources of variability from these steps. In DL techniques, multiple layers of neural networks, with varying modules (convolution/pooling) and activation functions, represent the data non-linearly. For example, the first layer might represent edges, the second can identify motifs in the edges, and the third can distinguish objects from the motifs.

Supervised DL methods such as CNN, patch-/pixel-based ML,^40^ and recurrent neural network^41^^,^^42^ are commonly used when ample labelled data are available. CNNs are popular in image recognition^43^ and are useful feature extractor layers of a DL architecture. In the convolutional layers of a CNN, the input image is divided into overlapping partial images through filters or kernels.^43^ Hence, the layers down-sample the image and extract semantic information until finally all the information is converted into target variables in the final layer.^19^ Alternatively, unsupervised methods can be used including autoencoders (AE)^44^ or recurrent Boltzmann machine methods.^5^ Semi-supervised approaches can combine supervised networks with unsupervised generative models for additional information about the image.^45^

DL methods are generally more flexible than hand-crafted feature selection and can be modified for various tasks including segmentation, registration, and lesion detection. For example, CNN trained on images of skin lesions resulted in dermatologist-level classification of skin cancer^46^ and a DL radiomics model using chest CT could predict distant metastasis.^47^ However, DL methods involve a larger number of parameters and a larger amount of data than hand-crafted methods.^48^ Data augmentation in DL can mitigate the issue of sparse data to some extent. Transfer learning can also be used to create a model trained on an unrelated dataset (eg, natural images) and use it on the target training dataset to fine-tune it to create a generalizable model on the target dataset.^5^^,^^49^

### Model development

#### Data pre-processing

When using hand-crafted radiomic features, variables that impact model training must be addressed including feature scaling, missing values, and class imbalance.^20^ Features scaling refers to normalization methods used to prevent features with greater ranges from dominating the model.^50^ Missing value is a common problem in real-world datasets and can be addressed by using simple statistics or complex modelling to impute the missing value.^20^ Class imbalance refers to the skewness of the dataset towards a certain classification label or predictive value (or range) and can be addressed by oversampling. Synthetic minority oversampling technique is used in oversampling to generate synthetic instances (feature sets) from real ones in the minority class.^51^ When using DL for modelling, data augmentation can be a useful approach to tackle class imbalance.

#### Feature classification

**Figure 3.**
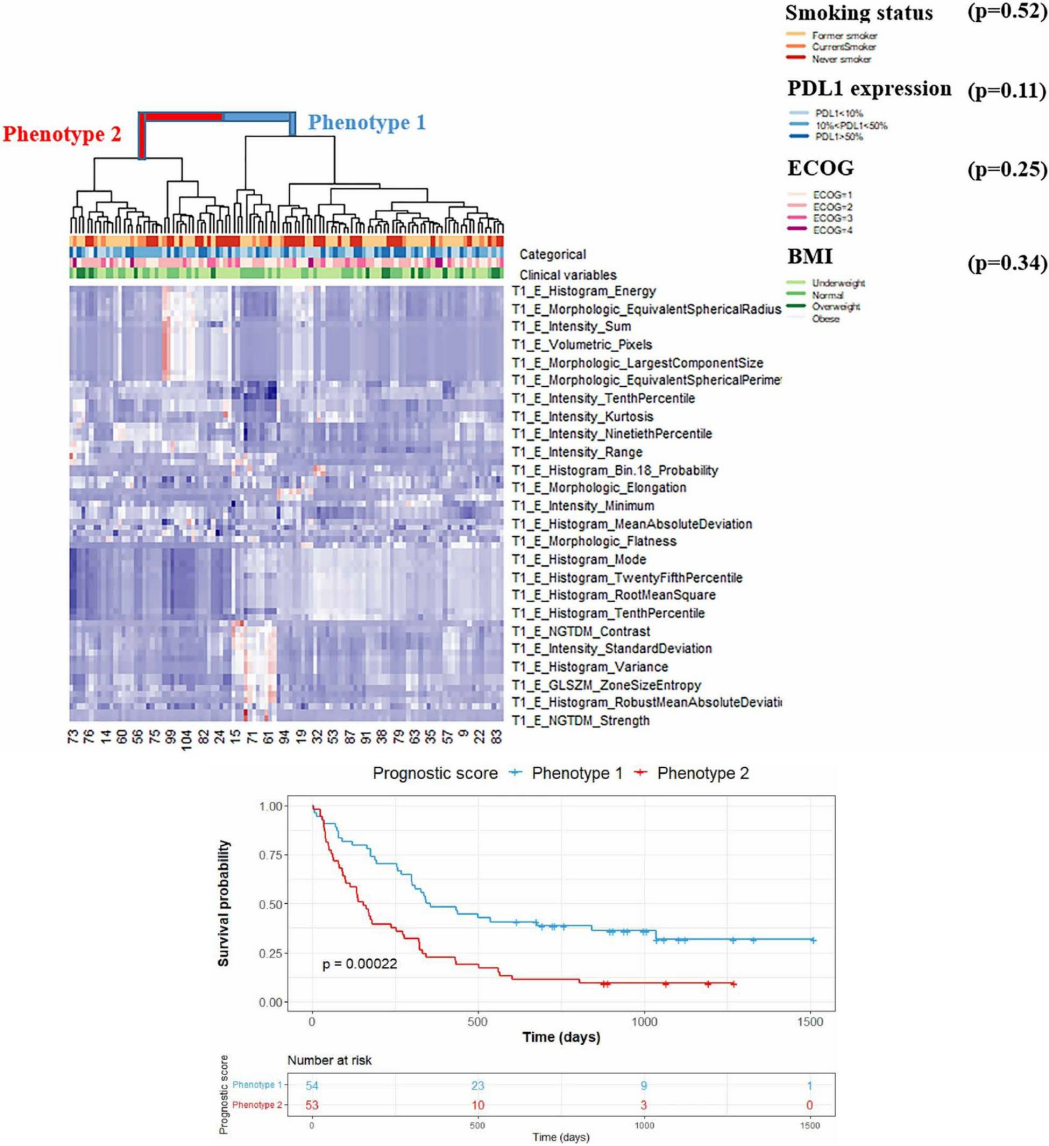
Hand-crafted radiomics feature modelling results from Singh et al.^22^ Top: Hierarchical clustering of radiomics features recognizes two statistically significant (*P* = .02) imaging phenotypes. Bottom: Survival analysis (using Kaplan-Meier curve). Progression-free survival probability for the two phenotypes using clinical covariates (PDL1 expression, Eastern Cooperative Oncology Group (ECOG), Body Mass Index (BMI), and smoking status) and radiomics phenotypes.

Once a set of non-redundant and robust features are curated, a model is built to answer the specific clinical question termed the “target variable.” Target variables can be discrete (ie, presence or absence of recurrence at 5 years), or continuous (ie, survival analysis). In the feature classification step of ML, the algorithm learns how to model the clinical question using examples of feature-target set (supervised ML) or by learning intrinsic patterns in the training feature set (unsupervised ML). The specific classification method is selected based on the medical question asked. Supervised classification algorithms, such as logistic regression,^52^ linear/non-linear support vector machine,^53^ random forest,^39^ Naïve Bayes,^37^ can be tested for discrete variables. For continuous target variables, linear regression and regression trees are valuable. Features can also be clustered into intrinsic imaging phenotypes using unsupervised clustering methods using an agglomerative approach for hierarchical clustering^22^^,^^54^ (Figure 3). For survival analysis, Kaplan-Meier curve,^22^^,^^55^ Cox regression,^56^ random survival forest,^49^ and support vector survival^46^ are typically used to explore variables or phenotypes that may impact survival time.

#### Model validation and evaluation

Once a radiomic model has been developed, it must then be validated. There are several different ways to validate radiomics models including quantification of predictive ability. Model validation is performed by separating the imaging data into training, validation, and testing sets. Usually, the training set is used during model building. Validation sets are used to fine-tune the ML setting (hyperparameters) to probe changes to the model’s performance. Sometimes, the training and validation are done on the same set and resampling techniques such as bootstrapping and cross-validation are used for validation.^5^^,^^20^ The test set is used to evaluate the performance of the now optimized model. To evaluate generalizability of the model, the test set can be curated from another scanner or site.

Several numerical metrics and graphical representations are used to evaluate performance. For binary classification, sensitivity (true positive rate) and specificity (true negative rate) are commonly used. The receiver operating characteristic (ROC) curve reveals the relationship between the true positive rate (y-axis) and the false-positive rate (x-axis). The area under the ROC represents the probability of the classifier randomly ranking a random positive instance higher than a random negative case. For survival analysis, c-statistics^47^ is used to evaluate the discriminative ability of the model. The Kaplan-Meier curve (Figure 3) is helpful for estimating survival among different clusters of imaging phenotypes^22^ and can be evaluated using the log rank test.^57^

## Applications of radiomics and AI in oncologic imaging

### Cancer screening and lesion detection

Multiple studies have shown a potential role for radiomics in the setting of optimizing lesion detection during cancer screening scans or routine clinical scans. For example, ML-based radiomic approaches have shown promise for distinguishing pancreatic ductal adenocarcinoma from normal pancreas in both diagnostic^58^ and prediagnostic^59^ CT scans and in detecting premalignant colorectal polyps using CT colonography.^60^ Radiomics has also shown promises in categorizing highly suspicious prostate cancer using multiparametric-MRI (mpMRI)^61–63^ and automated segmentation of tumour subregions.^64^ Other tissue-detection studies have been done on organs-at-risk contouring for radiotherapy using DL methods.^65^^,^^66^ By using these radiomic approaches, the sensitivity and specificity of lesion detection may be enhanced.

### Tissue characterization

Radiomics has also shown promise for predicting tissue histology including differentiating between benign and malignant lesions. If successful, this approach could assist in characterization of lesions that are in a location difficult to biopsy or during longitudinal follow-up of cancer patients to reduce the need for repeat biopsies. For example, DL (multiparametric magnetic resonance transfer learning) has been demonstrated to learn discriminative imaging features and categorize prostate cancers.^67^ Radiomics has been shown to successfully differentiate between benign and malignant lesions on mammography, ultrasound, and DCE-MRI images^68^^,^^69^ and distinguish different types of breast cancers.^52^^,^^53^ Radiomic features were also shown to predict the histology of lung tumours on CT.^37^^,^^70^ With this technology comes the potential of a “virtual biopsy” performed on lesions while still in situ which alleviates sampling error arising from internal tissue heterogeneity of tumours.^71^

### Prediction of tissue microenvironment and its effect on response

In addition to predicting tissue histology, radiomics-derived features have been demonstrated to predict tumour genomic expression characteristics and aspects of the tumour microenvironment. This is possible since the genomic expression and dynamics of the tumour microenvironment resulting in tissue structural changes that are detectable by radiomics. Aerts et al^31^ has developed prognostic radiomic signatures from lung and head-and-neck cancer (HNC) reflective of intratumoral heterogeneity and genomic expression. Radiomics signatures have been described that are associated with tumour features such as the degree of tumour mutational burden and presence of tumour-infiltrating lymphocytes both considered to be predictive of tumour response to anti-PDL1/PD1 therapy.^72^

Peritumoral tissues features can also be of predictive value, for example, on PET imaging in non–small-cell lung cancer (NSCLC) and cervical cancer.^73^ Since radiomics can detect these subtle tissue architectural features, radiomic signatures have been constructed that can predict oncologic therapy response. For example, radiomic can be leveraged to predict hypoxia in HNC patients, stratifying patients who would respond well to chemoradiotherapy based on tissue hypoxia.^74^ Another study has shown that spatial heterogeneity in MRI of glibliobastoma tumours is associated with 12-month overall survival and altered gene expression patterns.^75^

### Characterization of local response to therapy and prediction of recurrence or metastasis

Radiomics can also be of value in therapy response analysis allowing for earlier determination of therapy response or failure, and discrimination between posttreatment changes and residual viable tumour. For example, delta radiomics features, or longitudinal study of features on serial imaging, was shown to be a good predictor of necrosis versus progression in brain metastasis after radiosurgery.^76^ A phenotype of tumour heterogeneity extracted from preoperative DCE-MRI could predict 10-year recurrence rate^77^ and radiomics models on CT images of NSCLC patients after radiotherapy^78^ can predict the development of distant metastasis. Hand-crafted features from mpMRI trained on artificial neural network successfully predicted different response groups after preoperative chemoradiation therapy for locally advanced rectal cancer.^79^ Another study used a combination of clinical information and CT scans to predict progression-free survival in patients with NSCLC undergoing first-line immunotherapy.^22^

## Barriers to clinical translation

### Variabilities in image acquired

Radiomic approaches seek to leverage imaging to quantify biological variations in tissue. Radiomic models must be generalizable to enable accurate phenotyping on new “unseen” data (to the model) to be viable for clinical translation. A major confounder to this purpose are non-biological variations in medical images related to technical variations from scanner variability (manufacturer, model, hardware, sampling rate),^12^ inconsistent adherence to imaging protocols,^12^^,^^80^ variability in image reconstruction algorithms,^81^^,^^82^ hybrid protocols,^6^ and radiomics processing software.^23^ This results in different features extracted even in the same patient when imaged at different sites. This makes the use of multi-institutional data for radiomics challenging^6^ which is a major caveat for the validation of radiomic models and eventual clinical translation.

### Feature redundancy

The feature extraction process can produce a large number of features noting that the Image Biomarker Standardization Initiative has standardized 169 radiomic features.^23^ Large number of features can lead to model overfitting while training and will weaken model performance on new patient data. Among extracted features, it is sometimes difficult to establish which are relevant, and heterogeneous feature selection methods (“Feature Selection”) can negatively impact the reproducibility of these features as biomarkers.

### Feature and model interpretability

Feature interpretability becomes an issue especially with higher-order features such as texture features (ie, GLRLM, GLCM), and features after using filters. “Deep” features extracted also suffer from issue of low interpretability. Deep learning models are essentially “black-boxes,” and their layers cannot be interpreted leading to scepticism as to the value of the model.

### Heterogeneity in segmentation methods

The use of different segmentation methods can produce different radiomics signatures from the same image. This issue most significantly impacts manual segmentation, particularly when there are multiple annotators involved.^14^^,^^83^ Semi-automatic segmentation, although reduces manual labour, can introduce variability through varying degrees of user input. In addition, differences in user decision-making of what to include in a VOI (ie, solid portions of tumour vs necrotic components) introduce variability. An example of this is whether or not to include the peritumoural regions when predicting disease outcomes,^73^^,^^84^ a decision which has not been standardized by the field. While automatic segmentation techniques avoid the variability associated with user input, these methods work well on homogenous tumours but are challenged by irregular and heterogenous tumours.^17^

### Impact of comorbidities and therapies on tumour architecture

The power of radiomic analysis lies in the ability to leverage the digital imprint of the tumour architecture and interactions with the microenvironment. However, this level of granularity in tissue characterization means that the impact of clinical variables, such as those imparted by existing comorbidities and therapies, becomes more important as variables impacting the radiomic signal. Even among oncologic therapies, there is an increasing number of drug classes each shutting down tumour growth through a different strategy, most recently immunotherapeutics which work by inciting inflammation within the tumour. Systemic therapy can be co-administered with other modalities such as radiation therapy which incite inflammation and DNA damage within the tissues in and around the site of disease.

Furthermore, each individual patient presents with individual alterations in specific organs that may contribute to even further heterogeneity in imaging appearance of lesions. For example, a lung cancer in a patient with advanced background emphysema versus in a nonsmoker. Similarly, hepatocellular carcinoma in patients with variable degrees of liver cirrhosis. These patient factors may limit the generalizability of a given prediction model and more research is needed to elucidate the effect of comorbidities and interventions on radiomic signatures.

### Sample size and lack of big data

The impact of these sources of variability can be mitigated with large datasets that include clinical and imaging data, preferably from multi-institutional data sets. Obtaining these data sets is fraught with logistical and medico-legal issues that so far have limited most radiomic research to smaller data sets and retrospective analysis, significantly smaller than the datasets leveraged in past computer vision breakthroughs.^85^ The need for “big data” is most pressing when developing radiomic models in populations that are inherently more heterogeneous (ie, multiple possible interventions, histologies, or patient populations).^86^

## Tools needed for clinical translation

### Automated segmentation of lesions

Radiomic models perform best with homogeneous methods of segmentations whether automatic or semi-automatic.^14^ Automatic segmentation, an active area of research, can save time in radiomics analyses which require large datasets with homogenous pre-processing. Some automatic segmentation methods in development include Hierarchical CNNs for breast tumours in DCE-MRI,^87^ GAN-based segmentation for liver and brain tumours^88^ and U-net-based methods.^89^^,^^90^ U-net are of particular interest since they do not require as many training samples^91^ as some of the other approaches, a limiting factor for DL in medical imaging.

### Multicentre collaboration

Multicentre collaboration is of paramount importance in creating generalizable clinically relevant radiomic models. Moreover, it is important to validate these imaging biomarkers in multicentre prospective clinical trials through multi-institutional collaboration between imaging scientists, radiologists, and medical oncologists. Centralized learning (CL) initiatives where multiple institutions share patient data at a centralized location and develop clinical models are commonly used in radiomics. However, CL fails to work for a large number of institutions, especially on a global scale, due to concerns such as privacy, data ownership, and technical challenges.^92^ Conversely, federated learning, where the parameters from ML models trained on different centres are aggregated to form a consensus model, offers a decentralized approach to multicentre collaboration.^92^^,^^93^

### Standardization and harmonization of workflow

Adherence to standardized imaging acquisition protocols and proper reporting of the reconstruction algorithms will be important for creating high-quality multicentre aggregate data, both in real-world imaging acquisition and clinical trials. IBSI^74^ recently recommended strict adherence to standardized radiomics workflows and proper reporting of the methods used in different steps of the pipeline to optimize outcome. Realistically however regional practice differences even within a single health system may limit optimal standardization. Merging multicentre studies for ML and statistical models can, therefore, lead to poor results. Hence, the development of harmonization for imaging acquisition differences is vital to maximize the efficiency of multicentre studies. This is being studied in both image and feature domains.^27^ Image domain harmonization includes image-processing techniques (“Image Segmentation”). Feature-level harmonization includes batch-effect corrections using methods such as ComBat.^27^^,^^29^ Further optimization of these methods would play a role in a faster integration of radiomics into clinical practice.

## Conclusions and future directions of the field

This review aims to provide an overview of the workflow of radiomics, its potential clinical uses and remaining challenges currently limiting integration in routine clinical practice. We aimed to also integrate a brief discussion of the relevant AI tools used in the field of radiomics while being aware of the limitations of tackling these vast subjects in a short review.

A future goal of radiomics is to seamlessly integrate into the clinical workflow and augment radiological interpretation through quantitative measures; to assist radiologists in providing high-quality imaging interpretations, not replace them. Radiomics has shown great promise not only in areas of screening, diagnosis, and prognosis but also in areas such as therapy response prediction and assessment. Machine-learning-/DL-assisted radiomics can give treating clinicians a better insight of disease heterogeneity, progression, and therapy response on an individual level and help build targeted treatments—a step towards precision medicine. This is particularly promising in areas for heterogeneous disease such as cancer. Hence, it is crucial for researchers, developers, and clinicians to work to bring this technology to the clinical realm.

## References

[1] Hofman MS , Hicks RJ. How we read oncologic FDG PET/CT. Cancer Imaging. 2016;16(1):35. 27756360 10.1186/s40644-016-0091-3 PMC5067887

[2] Finelli PF. Diagnostic approach to restricted-diffusion patterns on MR imaging. Neurol Clin Pract. 2012;2(4):287-293. 30123680 10.1212/CPJ.0b013e318278bee1 PMC5829469

[3] Hamet P , Tremblay J. Artificial intelligence in medicine. Metabolism. 2017;69S:S36-S40. 28126242 10.1016/j.metabol.2017.01.011

[4] Morgan MB , Mates JL. Applications of artificial intelligence in breast imaging. Radiol Clin North Am. 2021;59(1):139-148. 33222996 10.1016/j.rcl.2020.08.007

[5] Avanzo M , Wei L, Stancanello J, et al Machine and deep learning methods for radiomics. Med Phys. 2020;47(5):e185-e202. 32418336 10.1002/mp.13678 PMC8965689

[6] Papanikolaou N , Matos C, Koh DM. How to develop a meaningful radiomic signature for clinical use in oncologic patients. Cancer Imaging. 2020;20(1):33. 32357923 10.1186/s40644-020-00311-4 PMC7195800

[7] Yushkevich PA , Piven J, Hazlett HC, et al User-guided 3D active contour segmentation of anatomical structures: significantly improved efficiency and reliability. Neuroimage. 2006;31(3):1116-1128. 16545965 10.1016/j.neuroimage.2006.01.015

[8] Fortin JP , Parker D, Tunc B, et al Harmonization of multi-site diffusion tensor imaging data. Neuroimage. 2017;161:149-170. 28826946 10.1016/j.neuroimage.2017.08.047 PMC5736019

[9] Johnson WE , Li C, Rabinovic A. Adjusting batch effects in microarray expression data using empirical Bayes methods. Biostatistics. 2007;8(1):118-127. 16632515 10.1093/biostatistics/kxj037

[10] Guiot J , Vaidyanathan A, Deprez L, et al A review in radiomics: making personalized medicine a reality via routine imaging. Med Res Rev. 2022;42(1):426-440. 34309893 10.1002/med.21846

[11] van Timmeren JE , Cester D, Tanadini-Lang S, Alkadhi H, Baessler B. Radiomics in medical imaging-"how-to" guide and critical reflection. Insights Imaging. 2020;11(1):91. 32785796 10.1186/s13244-020-00887-2 PMC7423816

[12] Li Y , Ammari S, Balleyguier C, Lassau N, Chouzenoux E. Impact of preprocessing and harmonization methods on the removal of scanner effects in brain MRI radiomic features. Cancers (Basel). 2021;13(12). 10.3390/cancers13123000 PMC8232807 34203896

[13] Song J , Zhang Z. Brain tissue segmentation and bias field correction of MR image based on spatially coherent FCM with nonlocal constraints. Comput Math Methods Med. 2019;2019:4762490. 30944578 10.1155/2019/4762490 PMC6421818

[14] Haarburger C , Muller-Franzes G, Weninger L, Kuhl C, Truhn D, Merhof D. Radiomics feature reproducibility under inter-rater variability in segmentations of CT images. Sci Rep. 2020;10(1):12688. 32728098 10.1038/s41598-020-69534-6 PMC7391354

[15] Gering D , Kotrotsou A, Young-Moxon B, et al Measuring efficiency of semi-automated brain tumor segmentation by simulating user interaction. Front Comput Neurosci. 2020;14:32. 32372938 10.3389/fncom.2020.00032 PMC7177174

[16] Ashraf AB , Gavenonis SC, Daye D, Mies C, Rosen MA, Kontos D. A multichannel Markov random field framework for tumor segmentation with an application to classification of gene expression-based breast cancer recurrence risk. IEEE Trans Med Imaging. 2013;32(4):637-648. 23008246 10.1109/TMI.2012.2219589 PMC4197832

[17] Rizzo S , Botta F, Raimondi S, et al Radiomics: the facts and the challenges of image analysis. Eur Radiol Exp. 2018;2(1):36. 30426318 10.1186/s41747-018-0068-z PMC6234198

[18] Parmar C , Rios Velazquez E, Leijenaar R, et al Robust radiomics feature quantification using semiautomatic volumetric segmentation. PLoS One. 2014;9(7):e102107. 25025374 10.1371/journal.pone.0102107 PMC4098900

[19] Cheng PM , Montagnon E, Yamashita R, et al Deep learning: an update for radiologists. Radiographics. 2021;41(5):1427-1445. 34469211 10.1148/rg.2021200210

[20] Stanzione A , Cuocolo R, Ugga L, et al Oncologic imaging and radiomics: a walkthrough review of methodological challenges. Cancers (Basel). 2022;14(19). 10.3390/cancers14194871 PMC9562166 36230793

[21] Lucia F , Visvikis D, Vallieres M, et al External validation of a combined PET and MRI radiomics model for prediction of recurrence in cervical cancer patients treated with chemoradiotherapy. Eur J Nucl Med Mol Imaging. 2019;46(4):864-877. 30535746 10.1007/s00259-018-4231-9

[22] Singh A , Horng H, Roshkovan L, et al Development of a robust radiomic biomarker of progression-free survival in advanced non-small cell lung cancer patients treated with first-line immunotherapy. Sci Rep. 2022;12(1):9993. 35705618 10.1038/s41598-022-14160-7 PMC9200843

[23] Zwanenburg A , Vallieres M, Abdalah MA, et al The image biomarker standardization initiative: standardized quantitative radiomics for high-throughput image-based phenotyping. Radiology. 2020;295(2):328-338. 32154773 10.1148/radiol.2020191145 PMC7193906

[24] Hatt M , Tixier F, Pierce L, Kinahan PE, Le Rest CC, Visvikis D. Characterization of PET/CT images using texture analysis: the past, the present… any future? Eur J Nucl Med Mol Imaging. 2017;44(1):151-165. 27271051 10.1007/s00259-016-3427-0 PMC5283691

[25] Aerts HJ. The potential of radiomic-based phenotyping in precision medicine: a review. JAMA Oncol. 2016;2(12):1636-1642. 27541161 10.1001/jamaoncol.2016.2631

[26] van Griethuysen JJM , Fedorov A, Parmar C, et al Computational radiomics system to decode the radiographic phenotype. Cancer Res. 2017;77(21):e104-e107. 29092951 10.1158/0008-5472.CAN-17-0339 PMC5672828

[27] Da-Ano R , Visvikis D, Hatt M. Harmonization strategies for multicenter radiomics investigations. Phys Med Biol. 2020;65(24):24TR02. 10.1088/1361-6560/aba798 32688357

[28] Orlhac F , Eertink JJ, Cottereau AS, et al A Guide to ComBat harmonization of imaging biomarkers in multicenter studies. J Nucl Med. 2022;63(2):172-179. 34531263 10.2967/jnumed.121.262464 PMC8805779

[29] Horng H , Singh A, Yousefi B, et al Generalized ComBat harmonization methods for radiomic features with multi-modal distributions and multiple batch effects. Sci Rep. 2022;12(1):4493. 35296726 10.1038/s41598-022-08412-9 PMC8927332

[30] Altman N , Krzywinski M. The curse(s) of dimensionality. Nat Methods. 2018;15(6):399-400. 29855577 10.1038/s41592-018-0019-x

[31] Aerts HJ , Velazquez ER, Leijenaar RT, et al Decoding tumour phenotype by noninvasive imaging using a quantitative radiomics approach. Nat Commun. 2014;5:4006. 24892406 10.1038/ncomms5006 PMC4059926

[32] van Timmeren JE , Leijenaar RTH, van Elmpt W, et al Test-retest data for radiomics feature stability analysis: generalizable or study-specific? Tomography. 2016;2(4):361-365. 30042967 10.18383/j.tom.2016.00208 PMC6037932

[33] Koo TK , Li MY. A guideline of selecting and reporting intraclass correlation coefficients for reliability research. J Chiropr Med. 2016;15(2):155-163. 27330520 10.1016/j.jcm.2016.02.012 PMC4913118

[34] Euler A , Laqua FC, Cester D, et al Virtual monoenergetic images of dual-energy CT-impact on repeatability, reproducibility, and classification in radiomics. Cancers (Basel). 2021;13(18):10.3390/cancers13184710 PMC8467875 34572937

[35] Baeßler B , Weiss K, Pinto Dos Santos D. Robustness and reproducibility of radiomics in magnetic resonance imaging: a phantom study. Invest Radiol. 2019;54(4):221-228. 30433891 10.1097/RLI.0000000000000530

[36] Balagurunathan Y , Kumar V, Gu Y, et al Test-retest reproducibility analysis of lung CT image features. J Digit Imaging. 2014;27(6):805-823. 24990346 10.1007/s10278-014-9716-x PMC4391075

[37] Wu W , Parmar C, Grossmann P, et al Exploratory study to identify radiomics classifiers for lung cancer histology. Front Oncol. 2016;6:71. 27064691 10.3389/fonc.2016.00071 PMC4811956

[38] Jovic A , Brkic K, Bogunovic N. A review of feature selection methods with applications. In: *2015 38th International Convention on Information and Communication Technology, Electronics and Microelectronics (MIPRO)*, Croatia, IEEE, 2015:1200-1205. doi: 10.1109/MIPRO.2015.7160458.

[39] Parmar C , Grossmann P, Bussink J, Lambin P, Aerts H. Machine learning methods for quantitative radiomic biomarkers. Sci Rep. 2015;5:13087. 26278466 10.1038/srep13087 PMC4538374

[40] Suzuki K. Pixel-based machine learning in medical imaging. Int J Biomed Imaging. 2012;2012:792079. 22481907 10.1155/2012/792079 PMC3299341

[41] Schmidt RM. *Recurrent Neural Networks (RNNs): A Gentle Introduction and Overview*. arXiv.org, 2019.

[42] Cho K , van Merrienboer B, Gulcehre C, et al Learning Phrase Representations using RNN Encoder–Decoder for Statistical Machine Translation. In: *Proceedings of the 2014 Conference on Empirical Methods in Natural Language Processing (EMNLP)*. Doha, Qatar. Association for computational linguistics, 2014:1724-1734.

[43] Lang N. Using convolutional neural network for image classification. Medium 2021. Accessed January 10, 2022. https://towardsdatascience.com/using-convolutional-neural-network-for-image-classification-5997bfd0ede4.

[44] Li F , Qiao H, Zhang B. Discriminatively boosted image clustering with fully convolutional auto-encoders. Pattern Recognition. 2018;83:161-173.

[45] Kingma PD , Rezende DJ, Mohamed S, Welling M. *Semi-supervised Learning with Deep Generative Models*. arXiv 1406.5298. 2014.

[46] Van Belle V , Pelckmans K, Van Huffel S, Suykens JA. Support vector methods for survival analysis: a comparison between ranking and regression approaches. Artif Intell Med. 2011;53(2):107-118. 21821401 10.1016/j.artmed.2011.06.006

[47] Uno H , Cai T, Pencina MJ, D'Agostino RB, Wei LJ. On the C-statistics for evaluating overall adequacy of risk prediction procedures with censored survival data. Stat Med. 2011;30(10):1105-1117. 21484848 10.1002/sim.4154 PMC3079915

[48] Bizzego A , Bussola N, Salvalai D, et al, Integrating deep and radiomics features in cancer bioimaging. In: *2019 IEEE Conference on Computational Intelligence in Bioinformatics and Computational Biology (CIBCB), July 9-11, 2019, Italy*. bioRxiv, 2019:1-8.

[49] Ishwaran H , Kogalur UB, Blackstone EH, Lauer MS. Random survival forests. Ann Appl Stat. 2008;2(3):

[50] Pedregosa F , Varoquaux G, Gramfort A, et al *Scikit-learn: Machine Learning in Python*. arXiv: 1201.0490, 2012.

[51] Chawla NV , Bowyer KW, Hall LO, Kegelmeyer WP. SMOTE: Synthetic minority over-sampling technique. JAIR. 2002;16:321-357.

[52] Lee SE , Han K, Kwak JY, Lee E, Kim EK. Radiomics of US texture features in differential diagnosis between triple-negative breast cancer and fibroadenoma. Sci Rep. 2018;8(1):13546. 30202040 10.1038/s41598-018-31906-4 PMC6131410

[53] Wang J , Kato F, Oyama-Manabe N, et al Identifying triple-negative breast cancer using background parenchymal enhancement heterogeneity on dynamic contrast-enhanced MRI: a pilot radiomics s. PLoS One. 2015;10(11):e0143308. 26600392 10.1371/journal.pone.0143308 PMC4658011

[54] Ward JH. Hierarchical grouping to optimize an objective function. J Am Stat Assoc. 1963;58(301):236-244.

[55] Bland JM , Altman DG. Survival probabilities (the Kaplan-Meier method). BMJ. 1998;317(7172):1572. 9836663 10.1136/bmj.317.7172.1572 PMC1114388

[56] Cox DR. Regression models and life-tables. J R Stat Soc Series B (Methodol). 1972;34(2):187-202.

[57] Schober P , Vetter TR. Kaplan-Meier curves, log-rank tests, and cox regression for time-to-event data. Anesth Analg. 2021;132(4):969-970. 33723194 10.1213/ANE.0000000000005358

[58] Chen PT , Chang D, Yen H, et al Radiomic features at CT can distinguish pancreatic cancer from noncancerous pancreas. Radiol Imaging Cancer. 2021;3(4):e210010. 34241550 10.1148/rycan.2021210010 PMC8344348

[59] Mukherjee S , Patra A, Khasawneh H, et al Radiomics-based machine-learning models can detect pancreatic cancer on prediagnostic computed tomography scans at a substantial lead time before clinical diagnosis. Gastroenterology. 2022;163(5):1435-1446 e3. 35788343 10.1053/j.gastro.2022.06.066 PMC12285712

[60] Grosu S , Wesp P, Graser A, et al Machine learning-based differentiation of benign and premalignant colorectal polyps detected with CT colonography in an asymptomatic screening population: a proof-of-concept study. Radiology. 2021;299(2):326-335. 33620287 10.1148/radiol.2021202363

[61] Stoyanova R , Takhar M, Tschudi Y, et al Prostate cancer radiomics and the promise of radiogenomics. Transl Cancer Res. 2016;5(4):432-447. 29188191 10.21037/tcr.2016.06.20 PMC5703221

[62] Khalvati F , Wong A, Haider MA. Automated prostate cancer detection via comprehensive multi-parametric magnetic resonance imaging texture feature models. BMC Med Imaging. 2015;15:27. 26242589 10.1186/s12880-015-0069-9 PMC4524105

[63] Algohary A , Viswanath S, Shiradkar R, et al Radiomic features on MRI enable risk categorization of prostate cancer patients on active surveillance: preliminary findings. J Magn Reson Imaging. 2018; 10.1002/jmri.25983 PMC6105554 29469937

[64] Li Q , Bai H, Chen Y, et al A fully-automatic multiparametric radiomics model: towards reproducible and prognostic imaging signature for prediction of overall survival in glioblastoma multiforme. Sci Rep. 2017;7(1):14331. 29085044 10.1038/s41598-017-14753-7 PMC5662697

[65] Ibragimov B , Xing L. Segmentation of organs-at-risks in head and neck CT images using convolutional neural networks. Med Phys. 2017;44(2):547-557. 28205307 10.1002/mp.12045 PMC5383420

[66] Lustberg T , van Soest J, Gooding M, et al Clinical evaluation of atlas and deep learning based automatic contouring for lung cancer. Radiother Oncol. 2018;126(2):312-317. 29208513 10.1016/j.radonc.2017.11.012

[67] Yuan Y , Qin W, Buyyounouski M, et al Prostate cancer classification with multiparametric MRI transfer learning model. Med Phys. 2019;46(2):756-765. 30597561 10.1002/mp.13367

[68] Antropova N , Huynh BQ, Giger ML. A deep feature fusion methodology for breast cancer diagnosis demonstrated on three imaging modality datasets. Med Phys. 2017;44(10):5162-5171. 28681390 10.1002/mp.12453 PMC5646225

[69] Sapate SG , Mahajan A, Talbar SN, Sable N, Desai S, Thakur M. Radiomics based detection and characterization of suspicious lesions on full field digital mammograms. Comput Methods Programs Biomed. 2018;163:1-20. 30119844 10.1016/j.cmpb.2018.05.017

[70] Ferreira Junior JR , Koenigkam-Santos M, Cipriano FEG, Fabro AT, Azevedo-Marques PM. Radiomics-based features for pattern recognition of lung cancer histopathology and metastases. Comput Methods Programs Biomed. 2018;159:23-30. 29650315 10.1016/j.cmpb.2018.02.015

[71] Dagogo-Jack I , Shaw AT. Tumour heterogeneity and resistance to cancer therapies. Nat Rev Clin Oncol. 2018;15(2):81-94. 29115304 10.1038/nrclinonc.2017.166

[72] Sun R , Limkin EJ, Vakalopoulou M, et al A radiomics approach to assess tumour-infiltrating CD8 cells and response to anti-PD-1 or anti-PD-L1 immunotherapy: an imaging biomarker, retrospective multicohort study. Lancet Oncol. 2018;19(9):1180-1191. 30120041 10.1016/S1470-2045(18)30413-3

[73] Hao H , Zhou Z, Li S, et al Shell feature: a new radiomics descriptor for predicting distant failure after radiotherapy in non-small cell lung cancer and cervix cancer. Phys Med Biol. 2018;63(9):095007. 29616661 10.1088/1361-6560/aabb5e PMC5963260

[74] Crispin-Ortuzar M , Apte A, Grkovski M, et al Predicting hypoxia status using a combination of contrast-enhanced computed tomography and [(18)F]-Fluorodeoxyglucose positron emission tomography radiomics features. Radiother Oncol. 2018;127(1):36-42. 29273260 10.1016/j.radonc.2017.11.025 PMC5924729

[75] Lee J , Narang S, Martinez JJ, Rao G, Rao A. Associating spatial diversity features of radiologically defined tumor habitats with epidermal growth factor receptor driver status and 12-month survival in glioblastoma: methods and preliminary investigation. J Med Imaging (Bellingham). 2015;2(4):041006. 26835490 10.1117/1.JMI.2.4.041006 PMC4718420

[76] Zhang Z , Yang J, Ho A, et al A predictive model for distinguishing radiation necrosis from tumour progression after gamma knife radiosurgery based on radiomic features from MR images. Eur Radiol. 2018;28(6):2255-2263. 29178031 10.1007/s00330-017-5154-8 PMC6036915

[77] Chitalia RD , Rowland J, McDonald ES, et al Imaging phenotypes of breast cancer heterogeneity in preoperative breast dynamic contrast enhanced magnetic resonance imaging (DCE-MRI) scans predict 10-year recurrence. Clin Cancer Res. 2020;26(4):862-869. 31732521 10.1158/1078-0432.CCR-18-4067 PMC7024654

[78] Huynh E , Coroller TP, Narayan V, et al CT-based radiomic analysis of stereotactic body radiation therapy patients with lung cancer. Radiother Oncol. 2016;120(2):258-266. 27296412 10.1016/j.radonc.2016.05.024

[79] Nie K , Shi L, Chen Q, et al Rectal cancer: assessment of neoadjuvant chemoradiation outcome based on radiomics of multiparametric MRI. Clin Cancer Res. 2016;22(21):5256-5264. 27185368 10.1158/1078-0432.CCR-15-2997 PMC10916000

[80] Cuocolo R , Stanzione A, Ponsiglione A, et al Prostate MRI technical parameters standardization: a systematic review on adherence to PI-RADSv2 acquisition protocol. Eur J Radiol. 2019;120:108662. 31539790 10.1016/j.ejrad.2019.108662

[81] Kim H , Park CM, Lee M, et al Impact of reconstruction algorithms on CT radiomic features of pulmonary tumors: analysis of intra- and inter-reader variability and inter-reconstruction algorithm variability. PLoS One. 2016;11(10):e0164924. 27741289 10.1371/journal.pone.0164924 PMC5065199

[82] Meyer M , Ronald J, Vernuccio F, et al Reproducibility of CT radiomic features within the same patient: influence of radiation dose and CT reconstruction settings. Radiology. 2019;293(3):583-591. 31573400 10.1148/radiol.2019190928 PMC10860741

[83] Joskowicz L , Cohen D, Caplan N, Sosna J. Inter-observer variability of manual contour delineation of structures in CT. Eur Radiol. 2019;29(3):1391-1399. 30194472 10.1007/s00330-018-5695-5

[84] Perez-Morales J , Tunali I, Stringfield O, et al Peritumoral and intratumoral radiomic features predict survival outcomes among patients diagnosed in lung cancer screening. Sci Rep. 2020;10(1):10528. 32601340 10.1038/s41598-020-67378-8 PMC7324394

[85] Varoquaux G , Cheplygina V. Machine learning for medical imaging: methodological failures and recommendations for the future. NPJ Digit Med. 2022;5(1):48. 35413988 10.1038/s41746-022-00592-y PMC9005663

[86] Willemink MJ , Koszek WA, Hardell C, et al Preparing medical imaging data for machine learning. Radiology. 2020;295(1):4-15. 32068507 10.1148/radiol.2020192224 PMC7104701

[87] Zhang J , Saha A, Zhu Z, Mazurowski MA. Hierarchical convolutional neural networks for segmentation of breast tumors in MRI with application to radiogenomics. IEEE Trans Med Imaging. 2019;38(2):435-447. 30130181 10.1109/TMI.2018.2865671

[88] Rezaei M , Nappi JJ, Lippert C, Meinel C, Yoshida H. Generative multi-adversarial network for striking the right balance in abdominal image segmentation. Int J Comput Assist Radiol Surg. 2020;15(11):1847-1858. 32897490 10.1007/s11548-020-02254-4 PMC7603459

[89] Haarburger C , Schock J, Truhn D, et al Radiomic feature stability analysis based on probabilistic segmentations. In *2020 IEEE 17th International Symposium on Biomedical Imaging (ISBI)*. 2020:1188-1192.

[90] Siddique N , Paheding S, Elkin CP, Devabhaktuni V. U-Net and its variants for medical image segmentation: a review of theory and applications. IEEE Access. 2021;9:82031-82057.

[91] Ronneberger O , Fischer P, Brox T. U-Net: Convolutional Networks for Biomedical Image Segmentation. In: *Medical Image Computing and Computer-Assisted Intervention—MICCAI*. Lecture notes in computer science (), vol 9351. Springer, Cham. 2015;234-241.

[92] Sheller MJ , Edwards B, Reina GA, et al Federated learning in medicine: facilitating multi-institutional collaborations without sharing patient data. Sci Rep. 2020;10(1):12598. 32724046 10.1038/s41598-020-69250-1 PMC7387485

[93] Tresp V , Marc Overhage J, Bundschus M, Rabizadeh S, Fasching PA, Yu S. Going digital: a survey on digitalization and large-scale data analytics in healthcare. Proc IEEE. 2016;104(11):2180-2206.

